# Does Nose Work Training Affect Dog Executive Function and Physical Fitness in Humans and Dogs?

**DOI:** 10.3390/ani16030453

**Published:** 2026-02-01

**Authors:** Heidi A. Kluess, Alexandra Hackett Neff

**Affiliations:** School of Kinesiology, Auburn University, Auburn, AL 36849, USA; alh0062@auburn.edu

**Keywords:** dog training, cognitive, scent work

## Abstract

Among domesticated animals, dogs are physically and behaviorally diverse. While centuries of breeding for specific traits accounts for many of these differences, myriad factors influence variation in cognitive and physical abilities among individual dogs. Here, we investigate whether training for nose work-type sports affects dog executive function and physical fitness in both dogs and their human companions. Given the demands of such training, we hypothesized that dogs and people who participate in nose work would have higher physical fitness, and the dogs would have advanced executive function compared to those that do not do the sport. Twenty-six dogs and seventeen human companions were recruited for this pilot study. Humans completed a questionnaire that included the Dog Executive Function Scale, information about dog training, and a 7-day physical activity recall. Humans and dogs performed a battery of fitness tests, and dogs completed two cognitive tests. We found that fitness scores did not differ between groups (nose work/non-nose work) in dogs or people, and nose work training associated with higher reported dog executive function scores. Notably, more formal nose work training was associated with less “giving up” in an unsolvable task, implying that training quality may impact task persistence in dogs.

## Simple Summary

Among domesticated animals, dogs are physically and behaviorally diverse. While centuries of breeding for specific traits accounts for many of these differences, myriad factors influence variation in cognitive and physical abilities among individual dogs. Here, we investigate whether training for nose work-type sports affects dog executive function and physical fitness in both dogs and their human companions. Given the demands of such training, we hypothesized that dogs and people who participate in nose work would have higher physical fitness and the dogs would have advanced executive function compared to those that do not do the sport. Twenty-six dogs and seventeen human companions were recruited for this pilot study. Humans completed a questionnaire that included the Dog Executive Function Scale, information about dog training, and a 7-day physical activity recall. Humans and dogs performed a battery of fitness tests, and dogs completed two cognitive tests. We found that fitness scores did not differ between groups (nose work/non-nose work) in dogs or in people, and nose work training was associated with higher reported dog executive function scores. Notably, more formal nose work training was associated with less “giving up” in an unsolvable task, implying that training quality may impact task persistence in dogs.

## 1. Introduction

Nose/scent work is a category of canine sport that involves using the dog’s nose to find and identify essential oils, animals (rats), shed (deer antlers), or human odors (handler discrimination/lost items, tracking, or mantrailing) under a variety of conditions, including in containers, indoors, and outdoors. For most scent/nose sports, the dog must search independently and interact with their human companion to let them know the location of the odor. These sports involve considerable cognitive energy from the dog and human to solve the odor puzzles [1] while walking over difficult terrain and often involve multiple searches in a day; therefore, the physical fitness of the person and the dog is important.

There is very little research into dogs and people who participate in scent/nose sports; however, these sports were originally developed to mimic activities performed by working dogs and their handlers in jobs like search and rescue and detection (drugs, electronics, explosives, etc). Therefore, it is likely that some of the same issues that affect the performance of working dog teams may also affect the performance of scent/nose work dog teams. Research in working dogs has demonstrated that physical stressors like poor physical fitness can reduce the ability of a dog to detect target odors [2]. Physical fitness may have the benefit of improving performance and also reducing the risk of injury in sport dogs [3,4,5]. For the purposes of this study, we selected fitness tests that were familiar to this lab [6] and appropriate for a wide range of ages of people, including the 6 min walk test, the chair sit-to-stand test, and the hand strength test. The 6 min walk test is a well-established fitness test for humans [7,8,9,10] that measures the distance covered in 6 min of walking as fast as possible. The chair sit-to-stand test is a measure of leg strength [11,12] and involves going from a sitting to a standing position and back to sitting as many times as possible in 30 s. This test is correlated with walking speed in older women [13]. The handgrip test is a common test of overall strength [14,15] and is performed using a handgrip dynamometer. Valid and reliable muscular fitness tests for physically active pet dogs are not well established, but there are some valid and reliable tests for older dogs [16,17]. There is work on cardiovascular endurance using treadmill exercise [18,19], but training dogs to walk on treadmills requires considerable time, and many never acquire the skill. The 6 min walk test [9,10] also has some precedent in pet dogs and requires no special skills. The Penn Vet Working Dog Center has piloted some foundational fitness tests for working dogs that may also be relevant for pet and sport dogs, including the squat test and the back-up test [20]. The squat test measures rear-end strength and the back-up test measures rear-end proprioception [20], which is important for traversing complex outdoor environments.

We know very little about cognitive processing in dogs that participate in sports; however, sport involvement typically involves considerable lifelong training. Some research suggests that dog training does improve cognitive function in dogs [21,22], but others suggest that there is no effect [23]. Previous research suggests that nose work training, specifically, improves dogs’ optimism regarding new things, compared to more traditional dog training [24]. Although not specifically a sport, dogs that do detection work [25,26] or participate in animal-assisted activities [27,28] score higher on cognitive tests. For this study we selected two cognitive tests: the object choice task [29,30] and the unsolvable task [31]. The object choice task involves the dog correctly interpreting pointing behavior from their person to correctly choose a bucket with a treat. This is a measure of social cognition that is important in nose work-type sports because the human and the dog have to navigate complex environments as a team. This test has been used successfully in pet dogs [32,33]. In the unsolvable task, the dog is presented with a treat that is encased in a clear box. The dog is given one minute to navigate the problem of the unattainable treat. The behavior exhibited by the dog is videoed and analyzed for the amount of time spent attending to the task, looking at the person, or avoiding the task/person. This is a measure of task persistence and social cognition (looking at their human companion). This test has been used to distinguish executive function parameters between dogs that participate in animal-assisted interventions and pet dogs [28] and breeds/breed groups of pet dogs [31,32].

For this pilot study, the working hypothesis is that human and dog teams that participate in nose work-type activities will have higher social cognition (object choice test), problem-solving ability (unsolvable task), and higher Dog Executive Function Scale (DEFS) scores [34] compared to dogs that do not participate in nose work-type activities. Since walking is part of nose work sports, we expect the nose work teams to have a higher 6 min walk distance and more reported moderate physical activity with the dog. Achievement in nose work sports generally involves considerable commitment to training; therefore, we expect the training rubric score (based on the amount of training and sport achievement) to be higher in the nose work dogs and the training rubric score to relate to the DEFS score, the executive function tests, the dog fitness tests, and the nose work level achieved.

## 2. Materials and Methods

### 2.1. Participants

This study was conducted in accordance with the Declaration of Helsinki and approved by the Institutional Review Board (protocol number 23-614 EP 2401, 11 January 2024) and the Institutional Animal Care and Use Committee (protocol number 2024-5379, 26 January 2024) of Auburn University, Auburn, AL, USA. We recruited teams (human and dog) that had nose/scent work dog training that met the following criteria:

1. Are self-taught in nose work;
2. Are or have been enrolled in an instructor-led nose work class;
3. Have completed any title in a nose work-type sport, including but not limited to those granted by the National Association of Canine Scent Work (NACSW), Barn Hunt Association, and American Kennel Club.

We also recruited dogs that have never participated in nose work-type training on their own or in an instructor-led class to serve as controls. These dogs and human companions were recruited through social media and flyers. Inclusion criteria for the humans were being able to walk with their dog and do some basic fitness tests (like those listed in this study). Inclusion criteria for the dog were to be at least 6 months old, treat-motivated, well-behaved enough to allow handling by the researchers, and up-to-date on vaccines. Dogs were excluded if they had current musculoskeletal injury, back/spine pain, hip dysplasia, osteoarthritis, or a significant knee injury. All testing took place at Auburn University in a temperature-controlled room with non-slip flooring. Testing was conducted in the order reported below.

### 2.2. Human Testing

#### 2.2.1. 6 Min Walk Test

The human and dog were asked to walk together as far as they could at a comfortable brisk pace for six minutes. The course was a quiet hallway with cones placed 43 m apart. They were encouraged to stop the test at any time if they or their dog needed to stop. The total distance was the score [7,8,9,10]. One researcher managed the timer and one researcher counted the laps.

#### 2.2.2. 30 s Chair Stand Test

The human was asked to complete a 30 s chair stand assessment in which the human sits in a chair (Vevor plyometric jump box, 18 inches, Vevor, Shanghai, China) and stands up as many times as possible in 30 s. The score was the number of repetitions completed [11,12]. One researcher managed the timer and one researcher counted the squats.

#### 2.2.3. Hand Strength

The human companion used a handgrip measurement device (T.K.K. 5001, Takei Grip Strength Dynamometer, Takei Scientific Instruments Co., Ltd., Tokyo, Japan) to measure dominant handgrip strength. The human squeezed the device as hard as they could. The researcher stopped them when the arrow on the dial stopped moving. The participant got three tries. One researcher wrote down the scores and conducted the test. The score was the average of the three tries [14,15].

### 2.3. Questionnaire

The human also filled out a questionnaire about themselves, their dog, their experiences with their dog, including involvement in nose work, current physical activity, dog training, and their dog’s executive function (Dog Executive Function Scale [34,35]; see questionnaire in Supplementary Materials). We assessed the amount of moderate and vigorous activity using the data from the 7-day physical activity recall in the questionnaire. Kilocalories of vigorous activity were calculated by the following equation: Kilocalories of vigorous activity = days per week × minutes per day × 7. The number 7 is the number of Kilocalories per min for vigorous activity according to the CDC [36]. Kilocalories of moderate activity were calculated by the following equation: Kilocalories of moderate activity = days per week × minutes per day × 3.5. The number 3.5 is the number of Kilocalories per min for moderate activity according to the CDC [36].

We used the questions about training to calculate a training rubric score (see Table 1). We developed this training rubric because we felt that previous research measuring training was not detailed and specific enough to address dog experiences in the USA. Marshall-Pescini et al. [37,38] defined no-training as requiring 10 lessons to learn basic commands and walking on a loose leash, while trained dogs included competition sport dogs and search and rescue dogs. In Range et al. [39], it was unclear how they determined training level, but they reported low training status as only basic training and high training as participating in training classes for activities such as obedience, agility, or rescue. Chapagain et al. [23,40] used a more rigorous method by asking dog companions about 13 types of training, including puppy school, obedience, agility, protection training, service dog training, herding, and therapy dog training, and they had companions score their dog’s experience/training, with no experience = 0, to complete training with/without an exam = 4. Our aim was to create something like Chapagain et al. [23], with an emphasis on the training and sport experiences common in the United States.

### 2.4. Dog Testing

#### 2.4.1. Cognitive Tests

Two cognitive tests have consistently shown promise in identifying dogs that excel in detection work in general [25,29,41]. Those tests are the unsolvable task and the object choice task. All cognitive tasks were recorded using a Go-Pro camera (Go-Pro Inc., San Mateo, CA, USA). For all tests, one researcher managed the dog and the second researcher managed the time and gave instructions to the dog’s companion.

#### 2.4.2. Unsolvable Task

The researcher lined the dog up at the start line (1.9 m from the box). The first several trials were warm-ups to get the dog comfortable with the unsolvable task apparatus. The human companion said, “Dog’s name, look!” and put a treat under the transparent box that was turned 45–90° to make it easy for the dog to push the box and retrieve the treat. The dog was released to retrieve the treat. Verbal encouragement was used if the dog seemed uncertain. This was repeated at least 4 times, making the angle of the box more challenging each time. For the unsolvable portion of the task, the researcher lined the dog up at the start position, and the human companion said, “Dog’s name, look!” and put a treat under the transparent box and locked the box in place. Once the human companion was back at their starting position, the researcher released the dog and allowed the dog to interact with the box for 1 min. Human companions were asked to stand in a neutral position and look up and away from the dog. No verbal or non-verbal encouragement was allowed. Videos were analyzed by a researcher blinded to group assignment for the amount of time spent interacting with the box, the amount of time gazing at the person, and the amount of time gazing away or walking away [31]. To assess inter-rater reliability, two independent coders scored the data, which were analyzed by a Pearson Product Moment correlation. The inter-rater reliability was *r* = 0.98 for the time spent interacting with the box, *r* = 0.99 for time spent looking at the human companion, and *r* = 0.97 for the time spent gazing or walking away, indicating excellent reliability.

#### 2.4.3. Object Choice Task

The buckets used in this task had a removable bottom with a small compartment. One treat was placed in the bottom compartment of each bucket to act as an odor control. For the warm-up portion of this task, the dog was positioned at the start line. The human companion was positioned between two buckets. The human companion placed a treat in the upper compartment of one bucket. The human companion said, “Dog’s name, look!” and pointed and looked at the baited container with one arm for approximately 2 s, then put their arm down. The human companion then said, “OK,” and the handler released the dog to make a choice. This was repeated several times on the left and right side. For the testing portion, the dog was removed from the room, and the human companion baited one container. The dog returned to the room and lined up at the start line (1.17 m from the human companion). The human companion said, “Dog’s name, look!” and pointed and looked at the baited container with one arm for approximately 2 s, then put their arm down. The human companion then said, “OK,” and the handler released the dog to make a choice. The dog had 60 s to make a choice. If the dog chose the correct container, they retrieved the treat. If the dog chose the wrong container, they were allowed to see that it was empty, and the dog was led outside the area for the next trial. Each dog did 10 trials. The outcome was the percent correct [29,30].

#### 2.4.4. Fitness Tests

The back-up and progressive squat tests were developed by the Penn Vet Working Dog Center Fit to Work Program [20]. We also performed a 6 min walk test and 2 measures of body condition.

#### 2.4.5. Back-Up Test

This test evaluated the dog’s body awareness. The human companion was asked to encourage their dog to back up 3 m 3 times in 30 s. If the dog was able to achieve this, then the dog was asked to back up 2.4 m and then up 2 steps (with rubber coating) that were placed to create a staircase (step 1: 18 cm tall × 30.5 cm in width; step 2: 30.5 cm tall × 28 cm wide). If the dog was able to reach the top step with their back feet, they repeated this again up to 3 times (time was limited to 30 s). The distance the dog was able to back up at all levels was recorded. One researcher managed the timer and one researcher gave instructions to the dog’s companion and recorded the distances.

#### 2.4.6. Progressive Squat Test

This test demonstrated rear-end strength. The dog sat on a textured mat in front of a stool that was at wither height (±2 inches) for the dog. The dog pushed up with its hindlegs and placed their front paws on the stool, then returned to the sit position. The human companion stood either behind or to the side of the stool and used treats to encourage the dog to perform the squat correctly. The researcher looked for a straight back and rear feet remaining in position on the mat. Any deviation resulted in an end to the test. The dog was asked to do 7 repetitions in 30 s. If the dog passed this level, the table height was increased to 1.5 times withers height and then 2 times withers height. The score was based on the total number of squats the dog successfully achieved with good form. One researcher managed the timer and one researcher counted the squats.

### 2.5. Body Condition Assessment

Body condition was measured by the pelvic circumference and hock-to-stifle length using a fabric tape measure, a standard technique in our lab [42,43]. One researcher measured the dog while the other researcher recorded the measurements. The anthropometric measurements were used to calculate the percent body fat of the dog using the following equations [44]:

Males % fat= −1.4 × (hock-to-stifle length^cm^) + 0.77 × (pelvic circumference^cm^) + 4

Females % fat= −1.7 × (hock-to-stifle length^cm^) + 0.93 × (pelvic circumference^cm^) + 54

We also did a Purina 9-point Body Condition Score evaluation on each dog.

### 2.6. Post Testing

The testing described above was repeated after 3 months.

### 2.7. Data Analysis

Data were reported as mean ± standard deviation. Differences between the nose work and non-nose work groups and among the non-nose work, self-taught, instructor-led, and titled groups were analyzed using a 1-way ANOVA with a Dunnett’s T3 post hoc test, if appropriate (SPSS version 29.0.0.0). Alpha was set a priori at 0.05. We also ran a Pearson Product Moment correlation on the cognitive, fitness, and nose work variables. To assess the reliability of fitness and cognitive variables over time, we ran a Pearson Product Moment correlation.

## 3. Results

### 3.1. Demographics of the Dogs and Humans

**Figure 1:**
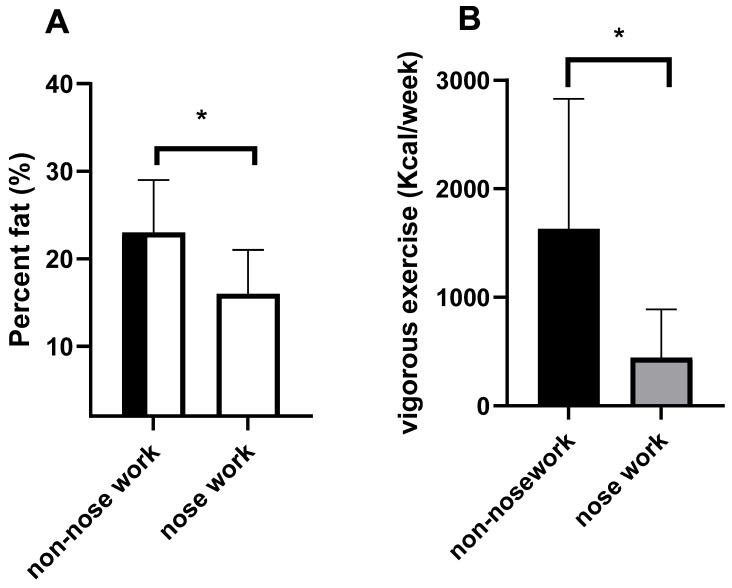
Summary of dog percent fat and vigorous exercise (as reported in the questionnaire). Dogs that participated in nose work training had a lower percentage of fat compared to non-nose work dogs (* *p* < 0.05). However, dog companions reported a higher amount of vigorous exercise in the non-nose work group compared to the nose work group (* *p* < 0.05).

We recruited 26 dogs (17 human companions). Seven dogs were in the non-nose work group and 19 dogs were in the nose work group. Breed information is provided in the Supplementary Materials (Table S1). The groups were well matched for dog age, but the percent fat (as measured by anthropometrics) was higher in the non-nose work group compared to the nose work group (F(1,25) = 7.43, *p* = 0.01, ETAsq = 0.237). See Figure 1A. However, The Purina Scale Body Condition values were not different (non-nose work: 5.6 ± 0.5; nose work: 5.5 ± 0.6). Human-companion-reported moderate exercise was similar between groups, but vigorous exercise was higher in the non-nose work group compared to the nose work group (F(1,17) = 24.39, *p* = <0.001, ETAsq = 0.504, see Figure 1B).

The dog demographic results are summarized in Table 2. Experience in dog sports, types of sports, titles, and nose work experience are summarized in Table 3. The training rubric score was not different between groups (F(1,22) = 1.67, *p* = 0.21).

The humans were younger in the non-nose work group (29 ± 8 years old), compared to the nose work group (45 ± 18 years old; F(1,24) = 4.8, *p* = 0.04, ETAsq = 0.41). The participants in both groups were predominantly female (nose work group: 1 male/18 females; non-nose work group: 2 males/5 females). The BMI for the nose work group (28 ± 5 kg/m^2^) was not different from the non-nose work group (21 ± 11 kg/m^2^).

### 3.2. Human Fitness

For the 6 min walk test, the person and dog walked together. There was no difference between the nose work group (507 ± 64 m) and the non-nose work group (546 ± 35 m). There were also no differences between the groups for the chair stand test (nose work group: 15 ± 4; non-nose work group: 18 ± 3 chair stands). The results of the handgrip test were not different (nose work group: 30 ± 8; non-nose work group: 38 ± 17 kg).

### 3.3. Dog Cognition

**Figure 2:**
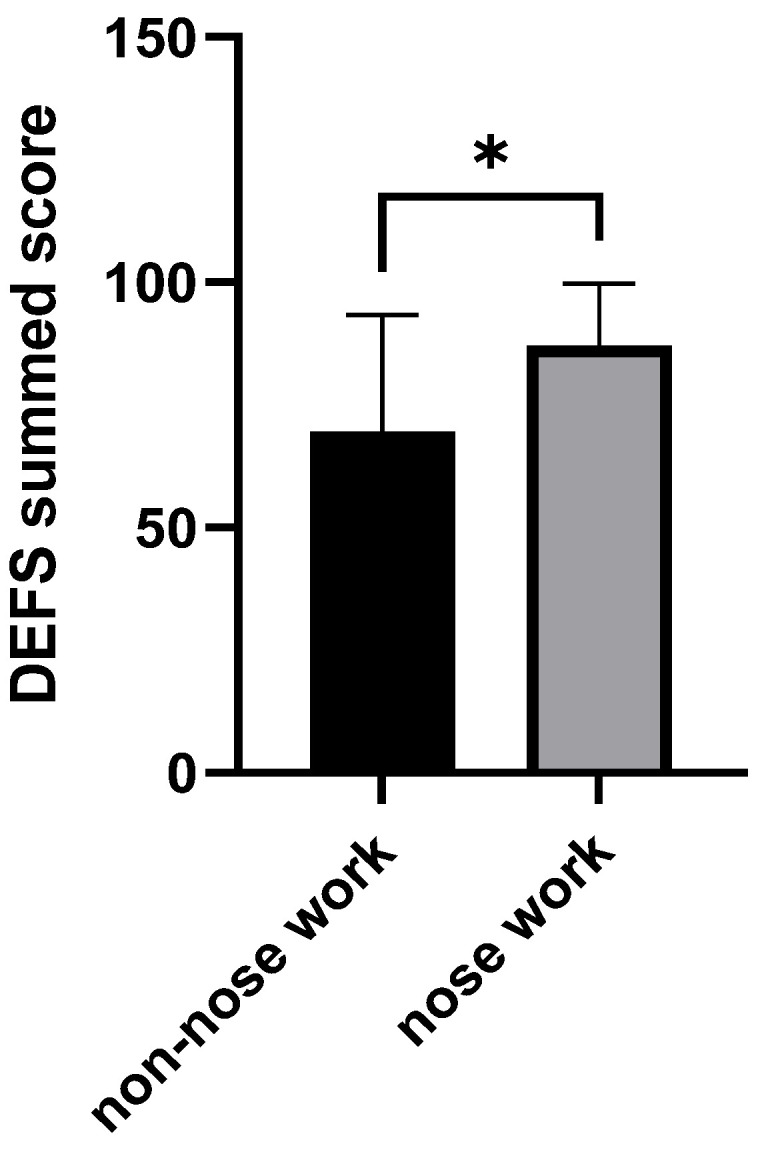
Summed score for the Dog Executive Function Scale (DEFS). Nose work-trained dogs scored higher on the DEFS than non-nose work dogs (* *p* < 0.05).

We measured executive function through the Dog Executive Function Scale (DEFS) and physical tests, including the object choice task and the unsolvable task. DEFS was higher in the nose work group than in the non-nose work group (F(1,24) = 5.91, *p* = 0.02, ETAsq = 0.198, see Figure 2).

**Figure 3:**
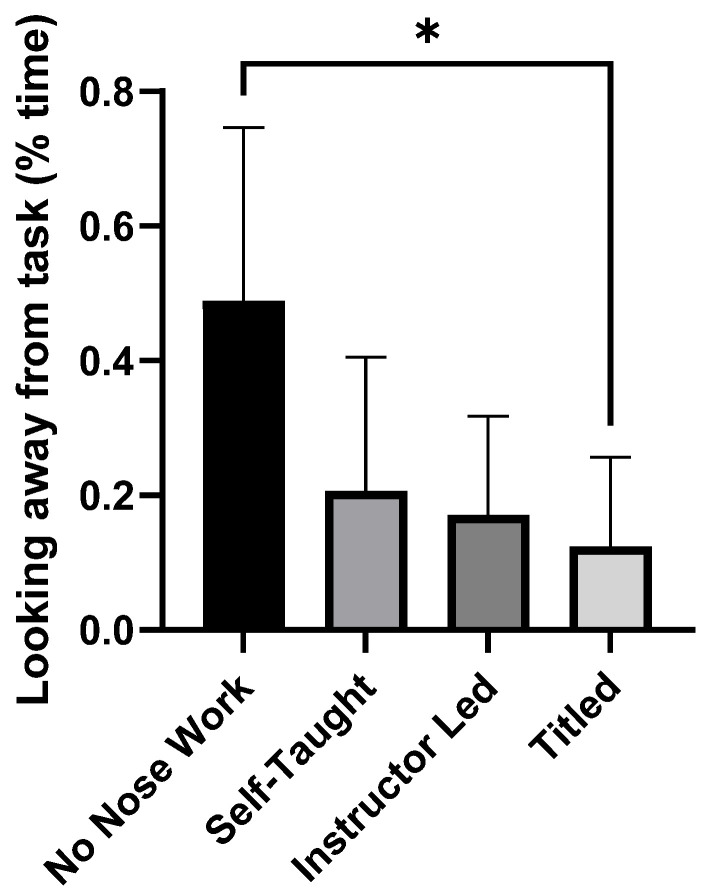
Percentage of time looking/gazing away from the task or human companion in the unsolvable task. The non-nose work group had a higher percentage of time spent gazing/looking away than the titled nose work group (* *p* < 0.05).

For the object choice task (social cognition), the non-nose work dogs were able to correctly identify the bucket with the treat 81 ± 25% of the time, and the nose work dogs identified the correct bucket 86 ± 17% of the time. For the unsolvable task, we analyzed videos for time spent attending to the task, looking at the human companion, and gazing or looking away. Attendance to the task (non-nose work: 36 ± 21; nose work: 59 ± 30% of time) and looking at the human companion (non-nose work: 17 ± 17; nose work: 22 ± 25% of time) were not different between the groups, but gazing or looking away occurred less in the nose work group (F(1,24) = 8.81, *p* = 0.007, ETAsq = 0.268). We also split the nose work group into a self-taught group (n = 6), an instructor-led group (n = 6), and a titled group (n = 6). Time spent looking away from the task/human companion was highest in the non-nose work group compared to the titled group (F(3,22) = 5.09, *p* = 0.008, ETAsq = 0.41), but was not different from the self-taught group or the instructor-led groups (see Figure 3).

### 3.4. Dog Fitness Tests

The non-nose work dogs were able to back up 3 ± 4 m, and the nose work dogs were able to back up 6 ± 4 m (F(1,24) = 3.19, *p* = 0.09). For the squat test, the nose work group was able to complete 5 ± 3 squats, and the non-nose work group completed 3 ± 1 squats (F(1,22) = 3.58, *p* = 0.07).

### 3.5. Correlations

We ran Pearson Product Moment correlations among the variables. We found that a higher nose work level (no nose work, self-taught, instructor-led, titled) was related to a lower percentage of time looking away in the unsolvable task (r= −0.577, *p* = 0.002) and lower vigorous exercise (r= −0.557, *p* = 0.003). A higher training rubric score and a higher DEFS score were also associated with a lower percentage of time looking away in the unsolvable task (training rubric: r= −0.449, *p* = 0.03; DEFS: r = −0.457, *p* = 0.02). The DEFS score was positively related to the amount of time the dog spent looking at the human companion in the unsolvable task (r = 0.533, *p* = 0.005).

For the human fitness tests, handgrip and vigorous exercise were positively correlated (r = 0.519, *p* = 0.007). The chair stand test and the 6 min walk test were also positively associated (r = 0.746, *p* = <0.001).

### 3.6. Reliability of Tests over Time

Twelve dogs (non-nose work = 5, nose work = 7) and 10 humans completed the fitness and cognitive testing again (5 ± 2 months from test 1 to test 2). Variables that were not significantly repeatable were percentage fat (dog: r = 0.358, *p* = 0.25, n = 12), percentage correct (object choice: r = 0.296, *p* = 0.35, n = 12), percentage of time spent attending to the human companion (unsolvable: r = 0.546, *p* = 0.067, n = 12), and percentage of time spent looking away (unsolvable: r = 0.200, *p* = 0.533, n = 12). Variables that had significant correlations from test 1 to 2 were the back-up test (r = 0.896, *p* = <0.0001, n = 12), the squat test (r = 0.717, *p* = 0.013, n = 11) the DEFS (r = 0.809, *p* = <0.001, n = 12), the percentage of time spent attending to the task (unsolvable: r = 0.779, *p* = 0.003, *p* = 12), the 6 min walk test (r = 0.780, *p* = 0.005, n = 11), the chair stand test (r = 0.870, *p* = <0.001, n = 11), and the handgrip test (r = 0.90, *p* = <0.001, n = 11).

## 4. Discussion

We found that Dog Executive Function Scale (DEFS) scores were higher in dogs that participated in nose work training than in non-nose work dogs. For the unsolvable task, non-nose work dogs spent much more time looking away from the task (giving up). When we split the groups by the type of nose work instruction they received, we found that the titled dogs had the lowest amount of time “giving up.” The self-taught and instructor-led groups were not different from the non-nose work group. This implies that the acquisition of a high level of nose work skill is important for improving persistence to a task. However, social cognition (object choice task) was not different between groups. Since walking is part of nose work sports, we expect the nose work teams to have a higher 6 min walk distance and greater reported moderate physical activity for the dog. We found no difference in the human or dog fitness tests or moderate exercise between the groups. However, teams that did nose work reported less vigorous activity, suggesting that they exchanged nose work training for vigorous activity. As expected, higher training rubric scores were related to higher DEFS scores and less time looking away in the unsolvable task, but not to dog fitness or to the nose work level achieved. This suggests that dog training, in general, may improve dog executive function and persistence in a task.

### 4.1. Fitness Tests

Overall, we found no differences in dog and human fitness between the groups. However, the non-nose work dogs were more over-fat compared to the nose work dogs, but the non-nose work dogs performed more vigorous physical activity with their companions than the nose work group. This finding is consistent with our previous research on body condition in sport versus pet dogs [42,43]. For the 6 min walk test, our participants walked with their dogs and walked an average of 517 m. This is in the upper percentile for women and much higher than for older women who were exercisers [45]. This was similar to healthy dogs that chose their own speed [10].

Our human participants ranged from 23 to 69 years old (average 41 years old); as a whole they were able to do an average of 15 chair stands in 30 s. This is well above the value that indicates a fall risk [36]. Handgrip strength was also not different between groups, but the average handgrip strength was 32 kg, which is a little higher than that reported for healthy women of similar age [14].

For dog fitness, we had dogs perform the progressive squat test and the back-up test based on the PennVet Working Dog Center Fit to Work Program [20]. There were no differences between the groups. To date, there are no fitness norms for pets or working dogs.

We also used a 7-day physical activity recall that asked about moderate and vigorous activity with their dog. The amount of moderate activity was not different between groups, but vigorous activity was about 1445 kcal per week less in the nose work group. We do not know why this is, but some people choose to participate in nose work sports because they are less physically demanding.

Overall, our participants (dog and human) had good fitness. Most participants reported some moderate and vigorous physical activity with their dog, and 69% reported a level of physical activity that met the guidelines of 500 kcal/week. Higher body fat in the non-nose work dogs is a concern, but the dogs were mostly overweight (Purina Body Condition Score of 6), not obese (Purina Body Condition Score of 7+). More work on human companions’ understanding of the correct body condition for dogs is needed.

### 4.2. Cognitive Tests

We measured executive function through the Dog Executive Function Scale (DEFS) developed by Foraita et al. [34] and two physical tests, the object choice task and the unsolvable task. The DEFS scores were higher in the nose work-trained group compared to the non-nose work-trained group. Foraita et al. [34,35] previously demonstrated a positive relationship between dog training scores and the DEFS. However, in our study, the training rubric score was not different between groups, suggesting that an association with nose work training itself may improve human-perceived executive function of their dog. The DEFS score was also positively correlated with the percentage of time spent looking at the human companion (unsolvable task). This indicates that dogs with higher companion-perceived executive function may have learned a positive association with seeking help from the human companion in a problem-solving task.

The Object Choice Task was not different between groups, suggesting similar levels of social cognition. The percent correct was similar to Junttila et al. [32] and Stewart et al. [33]. However, the percentage of time spent looking away in the unsolvable task was different between groups. We also analyzed the type of training the nose work groups received because we felt that more formal training might be superior to self-training. We found that the nose work titled teams had the lowest percentage of time spent looking away from the task compared to the other groups. This suggests that persistence for the task is an element of higher-level achievement in nose work training. Although we did not ask about the training of the instructor, the core principles of nose work training are teaching the dog to search independently of their handler and to be persistent in the search [46]. Overall, these results suggest that nose work training may have some unique cognitive advantages over other training types. This could be further tested by comparing high-achieving nose work teams to high-achieving agility teams. Both sports include high levels of training and persistence to the task, but nose work is primarily dog-led, and agility is human-led. Another future study could consider following nose work-trained dogs over time to see if nose work training can reduce cognitive decline with aging.

### 4.3. Reliability of Measures over Time

In a subset of dogs and humans, we repeated these tests after 3–6 months. We found that the dog back-up test, the dog squat test, the DEFS, the attendance to task (unsolvable), and the human fitness tests were reasonably reliable.

### 4.4. Limitations

One limitation of this study is the small sample size. This was a pilot study on the effects of nose work on cognition and physical fitness, and it is important to consider these conclusions carefully. However, the results suggest that there should be more studies in larger groups of participants. The idea that nose work may have some unique benefits compared to other types of dog training is an important issue.

We also potentially have some selection bias since we took all people who met our criteria and expressed interest in the study. We did have a small number of high-achieving nose work dogs in this study. Many of our participants were in the learning process for nose work and had not yet competed. While competition is not required, it does show a level of commitment and skill on the part of that team. As a side note, all of our self-taught and instructor-led participants went on to compete successfully in nose work competitions.

Cognitive measures like the Dog Executive Function Scale provide important insight regarding the human’s view of their dog’s executive function. It is important to continue to develop measures that take the human perspective out of the equation when evaluating executive function in dogs.

Lastly, fitness testing in pet dogs is in its infancy, and there are little data to compare. This was the first study, to my knowledge, attempting to use strength testing in physically active adult pet dogs. This is a very important fitness and health parameter for dogs and should continue to be explored.

## 5. Conclusions

In conclusion, we found that nose work training was associated with higher human-perceived executive function scores. However, the training rubric score was not different between groups. This suggests that nose work training may be associated with an increase in human-perceived executive function of their dog. More formal nose work achievement was associated with the least amount of “giving up” in the unsolvable task. This implies that the nose work skill level achieved is important for persistence to a task. Human and dog fitness scores were not different, but our participants scored at or above average in the fitness tests.

## Figures and Tables

**Table 1:** Training rubric.

Training Experience	Point Value
Human companion’s estimated training level	Multiply by 2 for point value
Each sport dog participates in with at least one title earned	1 point for each
Each sport dog participates in with no titles earned	0.5 point for each
Each listed title earned	0.5 point for each
Any CH (champion)-level title (MACH, RACH, ADCH, OTCH, etc.)	2 points each
Dog knows the behaviors: sit, down, shake, walk on leash	0.25 point each
Dog knows any other behaviors listed	0.5 point each
Experience with puzzle/mental stimulation type toys	0.25 point for “barely experienced: 0.5 point for “somewhat experienced” 1 point for “experienced” or “very experienced”
Each life stage indicated where dog attended any formal professional training	0.5 point
Clicker training	1 point
Sum = training level score	

**Table 2:** Dog group demographics.

	Non-Nose Work Group (n = 7)	Nose Work Group (n = 19)
Dog age	4 ± 2 years	4 ± 3 years
Dog sex Male (M)/Female (F)	M = 4/F = 3	M = 10/F = 9
Intact (I)/desexed (D)	I = 1/D = 6	I = 5/D = 14
Purebred (P)/mixed breed (mix)	P = 5/Mix = 2	P = 15/Mix = 4
Moderate exercise	480 ± 358 kcal/week	541 ± 676 kcal/week

**Table 3:** Training and experience.

	Non-Nose Work Group (n = 7)	Nose Work Group (n = 19)
Training rubric	12 ± 7	27 ± 9
Years in dog sports (Human)	Never competed n = 2 Less than year n = 3 1–3 years n = 2	Never competed n = 0
		Less than year n = 2
		1–3 years n = 7
		3–5 years n = 2
		5–7 years n = 3
		10+ years n = 5
Sports competed	Never competed n = 3	Never competed n = 0
	Obedience n = 1	Obedience n = 4
	Rally obedience n = 2	Rally obedience n = 5
	Herding n = 0	Herding n = 1
	Dock diving n = 3	Dock diving n = 9
	Agility n = 0	Agility n = 4
	Barn Hunt n = 0	Barn Hunt n = 7
	Scent Work n = 0	Scent Work n = 10
	IPO n = 0	IPO n = 1
	FastCat n = 1	FastCat n = 11
	Lure Coursing n = 1	Lure Coursing n = 1
	Conformation n = 1	Conformation n = 2
	Trick Dog n = 0	Trick Dog n = 11
	Disc sports n = 1	Disc sports n = 4
	Other n = 2	Other n = 2
Titles	n = 5 sports with titles	n = 40 sports with titles
Nose/scent work currently enrolled in classes	none	Yes n = 10/No n = 9
Years training in nose/scent work with an instructor	none	5 years n = 2
		3 years n = 2
		1–2 year n = 3
		less than 1 year n = 4
		none n = 8
Hours per week with an instructor	none	1.25 ± 0.50 h per week, n = 12 None = 7
Did they do informal practice	none	Yes n = 18, No n = 1
Total years of nose work practice (Human; can be multiple dogs)	none	5+ years n = 3
		3–4 years n = 3
		1–2 years n = 3
		less than 1 year n = 9
		no answer n = 1
Years the current dog trained in nose/scent work	none	4–5 years n = 2
		1–3 years n = 6
		Less than 1 year n = 11
Current nose/scent work titles	none	AKC excellent n = 5
		NACSW NW3 n = 1
		AKC advanced n = 5
		NACSW NW2 n = 1
		NACSW NW1 n = 3
		AKC novice n = 5
		NACSW ORT n = 3
		Other related titles n = 6
		No titles n = 13

NACSW = National Association of Canine Scent Work. AKC = American Kennel Club. ORT = odor recognition test. NW1–3 = nose work title level 1–3.

## Data Availability

The data are available at https://hak0006.wixsite.com/vascularphyslab/data.

## Supplementary Materials

The following supporting information can be downloaded at https://www.mdpi.com/article/10.3390/ani16030453/s1, Table S1: Breeds in each group; Questionnaire, Flyer for recruitment.
