# Supplementary material for: Does Nose Work Training Affect Dog Executive Function and Physical Fitness in Humans and Dogs?
Source: Animals (Basel). 2026 Feb 1;16(3):453. doi: 10.3390/ani16030453 (PMC12896905; doi:10.3390/ani16030453)
Supplement: Supplementary file 1 [file animals-16-00453-s001.zip › animals-4050971-supplementary.pdf]

Table S1: Breeds in each group

Non-nose work group	Nose work group
Mixed breed=2	Mixed breed=4
American Pit Bull Terrier=1	Border Collie=3
American Staffordshire Terrier=1	Chesapeake Bay Retriever=2
French Bulldog=1	English springer spaniel=1
	German Shepard Dog=1
Golden Retriever=2	Golden Retriever=1
	Labrador Retriever=3
	Parson Russell Terrier=1
	Poodle=1
	Standard Schnauzer=2

SECTION A: QUESTIONS ABOUT YOU AND YOUR FAMILY

A1. Are you? *(tick)*

1 ☐ Male
2 ☐ Female

A2. How old are you? _________ years

A3. What is your *household* annual income from all sources, before taxes? *(tick)*

Income Range	Tick
Less than $30,000	☐ 1
$30,001 to $50,000	☐ 2
$50,001 to $70,000	☐ 3
$70,001 to $90,000	☐ 4
$90,001 to $120,000	☐ 5
$120,001 to $150,000	☐ 6
$150,001 to $200,000	☐ 7
$200,001 plus	☐ 8

A4. What is your *highest* level of education? *(tick)*

1 ☐ Primary School
2 ☐ Secondary School
3 ☐ TAFE College
4 ☐ University (undergraduate degree)
5 ☐ University (post-graduate degree)
6 ☐ Other (write) ________________
7 ☐ No formal schooling

A5. How many people of the following ages *regularly* live in your household? *(write)*

Age	Number
i. Under 2 years	_____
ii. 2 – 6 years	_____
iii. 7 – 12 years	_____
iv. 13 – 17 years	_____
v. 18+ years	_____

A6. How tall are you? __________in
How much do you weigh? __________lbs

A7. How old is your dog? *(write)*

____

A8. What sex is your dog? *(tick)*

1 ☐ Male
2 ☐ Female

A9. Is your dog de-sexed? *(tick)*

1 ☐ Yes
2 ☐ No

A10. Is your dog a pure breed? *(tick)*

1 ☐ Yes
2 ☐ No

A11. If yes, what breed? *(write)*

____

A12. If no, what breed does he/she most resemble? *(write)*

____

A13. What size is your dog? *(tick)*

1 ☐ Extra small (e.g., toy poodle)
2 ☐ Small (e.g., Jack Russell)
3 ☐ Medium (e.g., Kelpie)
4 ☐ Large (e.g., Labrador)
5 ☐ Extra large (e.g., St Bernard)

A14. What do you use to evaluate your dog's body condition?

1 ☐ veterinarian
2 ☐ friends
3 ☐ dog trainer
4 ☐ articles in a magazine or on the internet
5 ☐ breeder

SECTION E: QUESTIONS ABOUT YOUR DOG'S SPORT PARTICIPATION

E1: How many years have you participated in dog sports? *(tick)*

1 ☐ less than 1 year
2 ☐ 1-3 yrs
3 ☐ 3-5yrs
4 ☐ 5-7yrs
5 ☐ 7-10yrs
6 ☐ 10+ yrs

E2: How many dogs are you currently competing with?

1 ☐ one dog
2 ☐ 2 dogs
3 ☐ 3 dogs
4 ☐ 4 dogs
5 ☐ 5+ dogs

E3: For the dog in this study, which sports does the dogs compete in?

1 ☐ obedience
2 ☐ rally obedience
3 ☐ herding
4 ☐ dock diving
5 ☐ agility
6 ☐ barn hunt
7 ☐ scent work
8 ☐ IPO
9 ☐ FastCat
10 ☐ Lure coursing
11 ☐ tracking
12 ☐ Earthdog
13 ☐ Flyball
14 ☐ Field trials
15 ☐ water trials
16 ☐ conformation
17 ☐ Trick dog
18 ☐ Disc dog/Frisbee sports
19 ☐ Other _______________

E4: For the dog in this study, which sports is he/she titled?

1 ☐ obedience
2 ☐ rally obedience
3 ☐ herding
4 ☐ dock diving
5 ☐ agility
6 ☐ barn hunt
7 ☐ scent work
8 ☐ IPO
9 ☐ FastCat
10 ☐ Lure coursing
11 ☐ tracking
12 ☐ Earthdog
13 ☐ Flyball
14 ☐ Field trials

15☐ water trials
16☐ conformation
17☐ Trick dog
18☐ Disc dog/Frisbee sports
19☐ Other _______________

E5: How many hours per week do you do sports specific training to prepare your dog for competition? *(tick)*

1 ☐ 0-1 hr per week
2 ☐ 1-3 hrs per week
3 ☐ 3-5 hrs per week
4 ☐ more than 5 hours per week

E6: How many hours a week do you spend on conditioning or foundation work that is not sport specific? For example, strength, stretching, foundation behaviors like body awareness, basic obedience? *(tick)*

1 ☐ 0-1 hr per week
2 ☐ 1-3 hrs per week
3 ☐ 3-5 hrs per week
4 ☐ more than 5 hours per week

E7: How many hours a week do you spend walking, running, fetch or biking with your dog? *(tick)*

1 ☐ 0-1 hr per week
2 ☐ 1-3 hrs per week
3 ☐ 3-5 hrs per week
4 ☐ more than 5 hours per week

Questions about nose/scent work activities

Are you a currently enrolled in instructor led classes for nose/scent work? Y N (circle one)

How many years have you been training for nose/scent work using instructor led classes? ______ years

How many hours per week do you do instructor led nose/scent work classes _______ hours/week

Do you do nose/scent work practices on your own or informally with others? Y N (circle one)
How many hours per week do you do nose/scent work practices on your own or informally with others? _______ hours/week

How many years have you done nose/scent work practices on your own or informally with others? _______ years

How many total years have you trained for nose/scent work (can be previous dogs) _______years

How many years has your current dog been training for nose/scent work? _______ years

For your current dog, what nose/scent work titles has your dog achieved to date?
____

<u>Questions about your dog and your activities with your dog</u>

Q80 What is the primary reason you own a dog?

- ◯ Pet/Companionship (1)
- ◯ Security (2)
- ◯ Service (3)
- ◯ Show/Sports (4)
- ◯ Hunting (5)
- ◯ Other: (6) ____

Q81 Where is your dog primarily kept?

- ◯ Inside (1)
- ◯ Outside (2)

Q82 How many *<u>days</u>* do you walk with your dog in a typical week? Dog walking is an activity in which both the dog and owner are walking together. The dog can be on or off a leash.

____

Q83 In *<u>minutes per walk</u>*, how much time to you spend walking during your typical dog walk?

____

Q84 On days you walk with your dog, on average how many walks do you go on?

- ◯ 1 walk (1)
- ◯ 2 walks (2)
- ◯ 3 walks (3)
- ◯ 4 walks (4)
- ◯ 5 or more walks (5)

Q109 Where do you most often walk your dog?

- ◯ Neighborhood (the area around your home, whether urban, suburban, or rural) (1)
- ◯ Public park (2)
- ◯ Isolated trails/open fields (3)

Q85 Do you have a yard for your dog?

- ◯ Yes (1)
- ◯ No (2)

Q86 If so, what is the approximate size of your yard?

- ◯ Small ((1)
- ◯ Medium (0.25-0.49 acre or 10,890-21,779 square feet) (2)
- ◯ Large (0.50 -0.99 acre or 21,780-43,560 square feet) (3)
- ◯ Very large (>1 acre or 43,560 square feet) (4)

Q87 Is your dog able to run freely in your yard?

- ◯ Yes (1)
- ◯ No (2)

Q88 Does your yard have a fenced area for your dog (physical fence or an electric fence)?

- ◯ Yes (1)
- ◯ No (2)

Q90 These questions are about your physical activity in the past 7 days. Please answer each question even if you do not consider yourself to be an active person. Think about the activities you do at work, your house and yard work, to get from place to place, and in your spare time for recreation, exercise or sport.

Q91 Think about all the vigorous activities which take hard physical effort that you did in the last 7 days. Vigorous activities make you breathe much harder than normal and may include heavy lifting, digging, aerobics, or fast bicycling. Think only about those physical activities that you did for at least 10 minutes at a time

Q92 How many days did you do vigorous physical activity in the last week? (answer in days/week)

____

Q93 How much time did you spend on vigorous physical activity these days (answer in min/day)

____

Q94 Now think about activities which take moderate physical effort that you did in the last 7 days. Moderate physical activities make you breathe somewhat harder than normal and may include carrying light loads, bicycling at a regular pace, or doubles tennis. Do not include walking. Again, think only about those physical activities that you did for at least 10 minutes at a time.

Q96 How many days did you do moderate physical activity in the last week? (answer in days/week)

____

Q97 How much time did you spend on moderate physical activity these days? (answer in min/day)

____

Q98 Now think about the time you spent walking in the last 7 days. This includes all walking at work and at home, walking to travel from place to place, dog walking and any other walking that you might do solely for recreation, sport, exercise, or leisure.

Q99 How many days did you do walk at least 10 minutes in the last week? (answer in days/week)

____

Q100 How much time did you spend walking on these days? (answer in min/day)

____

Q103 What factors encourage you to walk with your dog? (check all that apply)

- ☐ My health (1)
- ☐ Lose weight (2)
- ☐ Maintain dog's weight (3)
- ☐ Energetic dog (4)
- ☐ My dog's health (5)
- ☐ Good weather (6)
- ☐ Reduce dog's weight (7)
- ☐ Maintain my weight (8)
- ☐ Dog's enjoyment (9)
- ☐ Large dog (10)
- ☐ Other: (11) ____

Q104 What factors discourage you to walk with your dog? (check all that apply)

- ☐ Cold weather (1)
- ☐ Hot weather (2)
- ☐ Rain (3)
- ☐ Snow (4)
- ☐ My health (5)
- ☐ Dog is old (6)
- ☐ Dog is wild (7)
- ☐ Dog's health (8)
- ☐ Lack of time (9)
- ☐ Small dog (10)
- ☐ Untrained dog (11)
- ☐ Dog difficult to control (12)
- ☐ More than one dog to walk (13)
- ☐ Difficult for me to walk (14)
- ☐ Other: (15) ____

Training – The following questions will be used to assess your dog's training level. Please answer them as completely as possible!

1. To the best of your ability, list all behaviors/tricks your dog is proficiently trained in (i.e. sit, down, stay, shake, roll over, weave poles, etc.):
____
____
____
____
____
____
____
____

2. Does your dog participate in any dog sports (i.e. agility, dock diving, flyball, etc.)? If yes, list each sport, how long the dog has been involved, and at what level they are currently trialing/competing.

Dog sports	Length of involvement (years)	Level currently competing	Titles

3. Does your dog have experience with clicker training?
   a. Yes
   b. No

Are you a professional trainer? YES NO

4. On a scale of 1-10 where 10 is perfectly trained and 0 is not trained at all, honestly estimate your dog’s training level. This is meant to estimate actual training level, not necessarily how well-behaved your dog is in your daily life. Use the following descriptions as a rough guideline:

   (10) Dog can recognize over 50 cues/commands and performs them proficiently 95% of the time or better without having to be asked multiple times or given hints. They are able to perform their skills in novel environments and without seeing immediate reinforcement.
   (8) Dog can recognize 25 or more cues/commands and performs them proficiently 80% of the time or better. Dog can usually work in novel environments and does not need constant toy/or food reward to stay focused.
   (5) Dog knows 10 or so different cues/commands and performs them proficiently at least 60% of the time. May struggle a little in novel environments.
   (3) Dog can perform 5 or so cues relatively proficiently, does well at home/in class but less well behaved in public, might not always listen without food or toy.
   (1) Dog knows no or less than 3 cues, does not usually respond unless you have food or a toy, almost never listens to commands.

   Circle your dog’s estimated training level: 1 2 3 4 5 6 7 8 9 10

5. What is your dog’s experience level with food-based enrichment/mental stimulation toys (i.e. Kongs filled with food, treat balls, food-dispensing toys, puzzle toys, snuffle mats, etc.)?
   a. Very experienced – exposed on a regular basis (more than 2-3x a week) and exposed to different variants/toys
   b. Experienced – exposed semi-regularly (every other week to once or twice a week) and exposed to a variety of toys
   c. Somewhat experienced – exposed sporadically (once every month or two) with some experience with a variety of toys
   d. Barely experienced – exposed <5 times during lifetime and only one or a few different types
   e. No experience at all

LIFESTAGE QUESTIONS

PUPPY-birth-6mo

6. Did your puppy attend any formal training (group classes or private lessons with a professional trainer)?
    a. Yes
    b. No
7. What types of classes did your puppy attend DURING THIS LIFE STAGE? Circle all that apply.
    a. Puppy group class
    b. Private lessons – away from home
    c. Private lessons – at your home
    d. Manners/Basic Obedience group class (not specific to puppies)
    e. Advanced obedience class
    f. Sport training (agility, rally, etc.)

8. If your puppy underwent any formal training (not including any training performed at home by you or another owner) during this time period, what was the...
    a. Number of weeks your dog attended formal training: ________________ weeks
    b. Total hours each week your dog was in class/training: ________________ hours/week

(for example, if you attended a 6 week group class at a pet store with your dog and each class lasted an hour, you would write 6 in the first blank and 1 in the second blank)

9. How much time did you spend at home (outside of a class environment or otherwise supervised by a professional trainer) actively training your dog? This includes any kind of active training with your dog involving treats or other positive reinforcement, or punishment (i.e. basic obedience training, trick training, etc.)
    a. Total number of weeks you trained your dog: __________________ weeks
    b. Total hours per week spent training dog: ______________________ hours/week

ADOLESCENT – 6 months of age up to 18 months of age

10. Did your dog attend any formal training (group classes or private lessons with a professional trainer)?
    a. Yes
    b. No

11. What types of classes did your dog attend DURING THIS LIFE STAGE? Circle all that apply.
    a. Puppy group class
    b. Private lessons – away from home
    c. Private lessons – at your home
    d. Manners/Basic Obedience group class (not specific to puppies)
    e. Advanced Obedience class
    f. Sport training (agility, rally, etc.)

12. If your dog underwent any formal training (not including any training performed at home by you or another owner) during this time period, what was the...
    a. Number of weeks your dog attended formal training: ________________ weeks
    b. Total hours each week your dog was in class/training: ________________ hours/week

    (for example, if you attended a 6 week group class at a pet store with your dog and each class lasted an hour, you would write 6 in the first blank and 1 in the second blank) As a reference, this life stage contains approximately 12 months/52 weeks.

13. How much time did you spend at home (outside of a class environment or otherwise supervised by a professional trainer) actively training your dog? This includes any kind of active training with your dog involving treats or other positive reinforcement, or punishment (i.e. basic obedience training, trick training, etc.)
    a. Total number of weeks you trained your dog: __________________ weeks
    b. Total hours per week spent training dog: ______________________ hours/week

14. What skills were you training with your dog at this time (i.e. sit, down, potty training, recall, shake, tricks, agility foundations, body awareness, loose leash walking, leave it, etc.)
    ____
    ____
    ____

YOUNG ADULT – 18 months of age up to 4 years of age

TRAINING

15. Did your dog attend any formal training (group classes or private lessons with a professional trainer)?
    a. Yes
    b. No

16. What types of classes did your dog attend DURING THIS LIFE STAGE? Circle all that apply.
    a. Private lessons – away from home
    b. Private lessons – at your home
    c. Manners/Basic Obedience group class (not specific to puppies)
    d. Advanced Obedience class
    e. Sport training (agility, rally, etc.)
        i. List any sports the dog took formal instruction for in this life stage: ____ ____

17. If your dog underwent any formal training (not including any training performed at home by you or another owner) during this time period, what was the...
    a. Number of weeks your dog attended formal training: _______________ weeks
    b. Total hours each week your dog was in class/training: _______________ hours/week

(for example, if you attended a 6 week group class at a pet store with your dog and each class lasted an hour, you would write 6 in the first blank and 1 in the second blank) As a reference, this life stage contains approximately 30 months and 130 weeks.

18. How much time did you spend at home (outside of a class environment or otherwise supervised by a professional trainer) actively training your dog? This includes any kind of active training with your dog involving treats or other positive reinforcement, or punishment (i.e. basic obedience training, trick training, etc.)
    a. Total number of weeks you trained your dog: _________________ weeks
    b. Total hours per week spent training dog: _____________________ hours/week

What skills were you training with your dog at this time (i.e. sit, down, potty training, recall, shake, tricks, agility foundations, body awareness, loose leash walking, leave it, etc.)
____
____

<u>ADULT – 4 years of age up to 7 years of age</u>

TRAINING

19. Did your dog attend any formal training (group classes or private lessons with a professional trainer)?
    a. Yes
    b. No

20. What types of classes did your dog attend DURING THIS LIFE STAGE? Circle all that apply.
    a. Private lessons – away from home
    b. Private lessons – at your home
    c. Manners/Basic Obedience group class (not specific to puppies)
    d. Advanced Obedience class
    e. Sport training (agility, rally, etc.)
        i. List any sports the dog took formal instruction for in this life stage: ____ ____

21. If your dog underwent any formal training (not including any training performed at home by you or another owner) during this time period, what was the…
    a. Number of weeks your dog attended formal training: ________________ weeks
    b. Total hours each week your dog was in class/training: ________________ hours/week

(for example, if you attended a 6 week group class at a pet store with your dog and each class lasted an hour, you would write 6 in the first blank and 1 in the second blank) As a reference, this life stage contains approximately 36 months and 156 weeks.

22. How much time did you spend at home (outside of a class environment or otherwise supervised by a professional trainer) actively training your dog? This includes any kind of active training with your dog involving treats or other positive reinforcement, or punishment (i.e. basic obedience training, trick training, etc.)
    a. Total number of weeks you trained your dog: __________________ weeks
    b. Total hours per week spent training dog: ______________________ hours/week

23. What skills were you training with your dog at this time (i.e. sit, down, tricks, sport training, etc.) ____ ____ ____

MATURE ADULT – 7 years of age up to 9 years of age

TRAINING

24. Did your dog attend any formal training (group classes or private lessons with a professional trainer)?
    a. Yes
    b. No

25. What types of classes did your dog attend DURING THIS LIFE STAGE? Circle all that apply.
    a. Private lessons – away from home
    b. Private lessons – at your home
    c. Manners/Basic Obedience group class (not specific to puppies)
    d. Advanced Obedience class
    e. Sport training (agility, rally, etc.)
        i. List any sports the dog took formal instruction for in this life stage: ____ ____

26. If your dog underwent any formal training (not including any training performed at home by you or another owner) during this time period, what was the…
    a. Number of weeks your dog attended formal training: _________________ weeks
    b. Total hours each week your dog was in class/training: _________________ hours/week

(for example, if you attended a 6 week group class at a pet store with your dog and each class lasted an hour, you would write 6 in the first blank and 1 in the second blank) As a reference, this life stage contains approximately 24 months and 104 weeks.

27. How much time did you spend at home (outside of a class environment or otherwise supervised by a professional trainer) actively training your dog? This includes any kind of active training with your dog involving treats or other positive reinforcement, or punishment (i.e. basic obedience training, trick training, etc.)
    a. Total number of weeks you trained your dog: ___________________ weeks
    b. Total hours per week spent training dog: _______________________ hours/week

28. What skills were you training with your dog at this time (i.e. sit, down, tricks, sport training, etc.)
____
____
____

<u>SENIOR – 9 years of age up to 12 years of age</u>

TRAINING

29. Did your dog attend any formal training (group classes or private lessons with a professional trainer)?
    a. Yes
    b. No

30. What types of classes did your dog attend DURING THIS LIFE STAGE? Circle all that apply.
    a. Private lessons – away from home
    b. Private lessons – at your home
    c. Manners/Basic Obedience group class (not specific to puppies)
    d. Advanced Obedience class
    e. Sport training (agility, rally, etc.)
        i. List any sports the dog took formal instruction for in this life stage: ____ ____

31. If your dog underwent any formal training (not including any training performed at home by you or another owner) during this time period, what was the...
    a. Number of weeks your dog attended formal training: _________________ weeks
    b. Total hours each week your dog was in class/training: _________________ hours/week

(for example, if you attended a 6 week group class at a pet store with your dog and each class lasted an hour, you would write 6 in the first blank and 1 in the second blank) As a reference, this life stage contains approximately 36 months and 156 weeks.

32. How much time did you spend at home (outside of a class environment or otherwise supervised by a professional trainer) actively training your dog? This includes any kind of active training with your dog involving treats or other positive reinforcement, or punishment (i.e. basic obedience training, trick training, etc.)
    a. Total number of weeks you trained your dog: ___________________ weeks
    b. Total hours per week spent training dog: _______________________ hours/week

33. What skills were you training with your dog at this time (i.e. sit, down, tricks, sport training, etc.)
    ____
    ____
    ____

Dog Executive Function Scale

Authors: Maike Foraita[1], Dr Tiffani Howell[1], Prof Pauleen Bennett[1]

1. Anthrozoology Research Group, School of Psychology and Public Health, La Trobe University, Melbourne, Australia

Please indicate to what extent the below statements apply to your dog. Some of these statements are very similar, so please read each one carefully. Please only tick 'not applicable' if you have never observed a situation like the one described in the statement. If you have more than one dog, please refer to the dog whose name starts with the letter closest to the letter 'A'.

Q		Never or almost never	Rarely	Sometimes	Often	Always or almost always	Not applicable
1	My dog gets upset about changes in the environment (e.g. a new piece of furniture).	☐	☐	☐	☐	☐	☐
2	My dog can relax in public places (e.g. a café).	☐	☐	☐	☐	☐	☐
3	My dog adapts well to new situations and environments.	☐	☐	☐	☐	☐	☐
4	My dog can relax in unfamiliar environments (e.g. a friend’s house, a holiday home).	☐	☐	☐	☐	☐	☐

		Never or almost never	Rarely	Sometimes	Often	Always or almost always	Not applicable
5	My dog gets excited around other dogs.	☐	☐	☐	☐	☐	☐
6	My dog gets over-excited about things and can be a bit "over the top" at these times.	☐	☐	☐	☐	☐	☐

7	Overall, my dog is excitable.	☐	☐	☐	☐	☐	☐
8	My dog needs constant reminding to control behaviours which are inappropriate (e.g. jumping up on visitors).	☐	☐	☐	☐	☐	☐

		Never or almost never	Rarely	Sometimes	Often	Always or almost always	Not applicable
9	I can easily get my dog's attention.	☐	☐	☐	☐	☐	☐
10	I can hold my dog’s attention for minutes at a time.	☐	☐	☐	☐	☐	☐
11	My dog gazes at me or turns toward me when I speak to him/her.	☐	☐	☐	☐	☐	☐

		Never or almost never	Rarely	Sometimes	Often	Always or almost always	Not applicable
12	My dog can follow an instruction for a minute (e.g. ‘sit’ or ’stay’).	☐	☐	☐	☐	☐	☐
13	My dog can follow an instruction (e.g. ‘stay’) in a quiet place (e.g. at home).	☐	☐	☐	☐	☐	☐
14	My dog will follow instructions (e.g. 'sit' or 'stay') when the cue is slightly different than normal (e.g. change in tone or pitch).	☐	☐	☐	☐	☐	☐
15	My dog will follow instructions (e.g. 'sit' or 'stay') given by a stranger.	☐	☐	☐	☐	☐	☐

		Never or almost never	Rarely	Sometimes	Often	Always or almost always	Not applicable
16	My dog finds it difficult to tolerate waiting for a reward.	☐	☐	☐	☐	☐	☐
17	My dog finds it difficult to tolerate waiting for a walk.	☐	☐	☐	☐	☐	☐
18	My dog finds it difficult to tolerate waiting for dinner.	☐	☐	☐	☐	☐	☐
19	My dog gets frustrated when he/she is not immediately rewarded for a behaviour.	☐	☐	☐	☐	☐	☐

		Never or almost never	Rarely	Sometimes	Often	Always or almost always	Not applicable
20	When playing, my dog easily gets distracted by other things.	☐	☐	☐	☐	☐	☐
21	It is difficult for my dog to concentrate on a single activity (e.g. chewing, playing).	☐	☐	☐	☐	☐	☐
22	My dog often forgets what he/she was doing after getting distracted (e.g. forgets about a toy or treat if a loud noise distracted him/her for a moment).	☐	☐	☐	☐	☐	☐
23	My dog forgets about something he/she wanted once it is out of sight (e.g. toy, food).	☐	☐	☐	☐	☐	☐

AUBURN UNIVERSITY

SCHOOL OF KINESIOLOGY

DO YOU OWN A DOG?

You are invited to participate in a research study to examine the long term fitness of dog owners and their dogs. We will ask you to participate in testing at least once per year for 3 years.

Eligibility criteria:

1. You own a dog at least 6 months old.
2. The dog must be treat motivated and well behaved enough to allow handling by the researchers.
3. The dog must be up to date on vaccines.
4. Dog cannot have a current musculoskeletal injury, back/spine pain, hip dysplasia, osteoarthritis, or a significant knee injury.

As a participant, you will be completing a questionnaire about your health and physical activity, your dog's health and physical activity and general information about you and your dog. You will also be asked to participate in fitness tests like getting out of a chair, measuring your grip strength and walking for 6 minutes. Your dog will also do some fitness tests and cognitive tests.

Your time commitment will be approximately 2 hours per visit, for a max of 3 visits over 3 years.

Benefits: Although there are no direct benefits to you, the researchers will use the results from this research to provide more information on the effects of dog ownership on fitness and physical activity.

If you have questions about this study or to participate, contact Dr. Heidi Kluess at hak0006@auburn.edu.

The Auburn University Institutional Review Board has approved this Document for use from 01/11/2024 to -------------- Protocol # 23-614 EP 2401

301 Wire Road, Auburn, AL 36849-5323; Telephone: 334-844-4483; Fax: 334-844-1467

www.auburn.edu
